# Vitamin D Status in Women with Gestational Diabetes Mellitus during Pregnancy and Postpartum

**DOI:** 10.1155/2015/260624

**Published:** 2015-04-27

**Authors:** Anna Pleskačová, Vendula Bartáková, Lukáš Pácal, Katarína Kuricová, Jana Bělobrádková, Josef Tomandl, Kateřina Kaňková

**Affiliations:** ^1^Department of Pathophysiology, Faculty of Medicine, Masaryk University, Kamenice 5, 625 00 Brno, Czech Republic; ^2^Department of Biochemistry, Faculty of Medicine, Masaryk University, Kamenice 5, 625 00 Brno, Czech Republic; ^3^Department of Internal Medicine, University Hospital Brno, Jihlavská 20, 625 00 Brno, Czech Republic

## Abstract

Of many vitamin D extraskeletal functions, its modulatory role in insulin secretion and action is especially relevant for gestational diabetes mellitus (GDM). The aims of the present study were to determine midgestational and early postpartum vitamin D status in pregnant women with and without GDM and to describe the relationship between midgestational and postpartum vitamin D status and parallel changes of glucose tolerance. A total of 76 pregnant women (47 GDM and 29 healthy controls) were included in the study. Plasma levels of 25(OH)D were measured using an enzyme immunoassay. Vitamin D was not significantly decreased in GDM compared to controls during pregnancy; however, both groups of pregnant women exhibited high prevalence of vitamin D deficiency. Prevalence of postpartum 25(OH)D deficiency in post-GDM women remained significantly higher and their postpartum 25(OH)D levels were significantly lower compared to non-GDM counterparts. Finally, based on the oGTT repeated early postpartum persistent glucose abnormality was ascertained in 15% of post-GDM women; however, neither midgestational nor postpartum 25(OH)D levels significantly differed between subjects with GDM history and persistent postpartum glucose intolerance and those with normal glucose tolerance after delivery.

## 1. Introduction

Diabetes mellitus with the first onset in pregnancy—a gestational diabetes mellitus (GDM)—is a common complication of pregnancy [1]. The frequency of GDM may reach up to 18% depending on the population and diagnostic criteria used [2]. Even the normal pregnancy is characterized by a marked reduction in maternal insulin sensitivity in the second and third trimesters. However, the reduced *β* cells reserve or their maladaptation to higher insulin demands may lead to the development of GDM. Resulting abnormal metabolic situation during GDM pregnancy might adversely influence the foetal development (resulting most often in macrosomia with subsequent delivery complications and possibly also the postnatal health status of offspring due to the foetal programming). Moreover, GDM is a significant predictor of woman's predisposition to the development of overt diabetes mellitus type 2 later in life as documented by epidemiological studies [3, 4]. In addition, GDM strongly predicts cardiovascular disease in the future life. The risk is increased by 70% in women with a previous history of GDM compared to women without this history [5].

Vitamin D has traditionally been viewed as a key regulator of bone mineralisation [6] and calcium homeostasis [7]; however, the documented effects are far more pleiotropic. Vitamin D facilitates active calcium absorption in the small intestine by increasing calcium channel and calcium binding protein expression. Furthermore, it interacts with its receptor in osteoblasts and promotes the maturation of preosteoclasts. Besides that, growing evidence mounted that vitamin D has a number of extraskeletal functions. Vitamin D—via its binding to the vitamin D receptor (VDR)—regulates expression of hundreds of genes (directly or indirectly) including those that control key processes affecting cell fate [8]. The complexity of vitamin D action is further increased by VDR gene polymorphism. The reported associations with plethora of phenotypes (including cancer, autoimmune, cardiovascular, metabolic, and renal and many other diseases) have been extensively meta-analysed and reviewed [9, 10]. In general, vitamin D decreases cell proliferation and stimulates cell maturation and apoptosis. Furthermore, vitamin D has a strong immunomodulatory effect; it inhibits angiogenesis [8] and is also involved in the regulation of insulin secretion and possibly insulin action [11, 12]. Interestingly vitamin D also exerts renoprotective and antiproteinuric effects with several mechanisms involved including inhibition of renin-angiotensin-aldosteron system (by decreasing renin expression), suppression of inflammation (by reducing accumulation of inflammatory cells), and restoration of glomerular filtration barrier (by attenuating podocyte damage) [13–15].

The major source of vitamin D is skin after sunlight exposure. Cutaneous vitamin D synthesis is modulated by several factors including skin pigmentation, clothing, melanin concentration, latitude, climate type, and season [16]. Vitamin D, either produced in the skin* de novo* from cholesterol (cholecalciferol) or ingested from the diet as a precursor (cholecalciferol and ergocalciferol), undergoes hydroxylation to 25-hydroxyvitamin D (25(OH)D) in the liver. Circulating plasma concentration of 25(OH)D is considered the most reliable indicator of individual's vitamin D status. 25(OH)D is further hydroxylated to the active 1,25-dihydroxyvitamin D (1,25(OH)_2_D) almost exclusively in the kidney upon regulation by parathormon [17]. Several studies have consistently shown that 1,25(OH)_2_D concentration increases progressively during gestation being twice as high in late pregnancy as in postpartum or in nonpregnant controls [17, 18]. The active form 1,25(OH)_2_D is also produced by placenta during pregnancy [19] with possible autocrine or paracrine function [20].

A number of studies focused on putative role of vitamin D deficiency in various pregnancy pathologies including GDM [21–23]. Observational studies revealed correlation between low vitamin D levels and preeclampsia or GDM [7]. Vitamin D deficiency in pregnancy was related to the incidence of GDM and serum 25(OH)D was significantly lower in women with GDM than in those with normal glucose tolerance [24–28]. Whether this association is causal remains however unclear [29]. Furthermore, several studies found inverse correlation between 25(OH)D and fasting plasma glucose (FPG), 1 hr after load plasma glucose in oral glucose tolerance test (oGTT) and glycated haemoglobin [30, 31].

Currently, little is known about postpartum vitamin D status in women with history of GDM and possible relationship between 25(OH)D plasma levels measured at the time of GDM diagnosis and the degree of glucose (in)tolerance postpartum. We hypothesise that individual's midgestational 25(OH)D plasma levels might independently reflect the risk of postpartum persistence or early reoccurrence of glucose abnormality in women with GDM history. Therefore, the aims of the present study were (1) to determine midgestational and early postpartum vitamin D status by measuring 25(OH)D plasma levels in pregnant women with and without GDM to confirm the hypothetical deficiency in GDM in central European population and (2) to describe the relationship between midgestational and postpartum vitamin D status and parallel changes of parameters characterising glucose tolerance.

## 2. Materials and Methods

### 2.1. Subjects

To avoid the confounding factor of seasonal variation of vitamin D levels, the recruitment of study subjects was confined to women whose 24–30th weeks of gestation (i.e., the first, midgestational blood sampling) spanned winter months (i.e., January, February, and March). Therefore, the inclusion criteria were (i) GDM or non-GDM diagnosed by 3-point oGTT between 24th and 30th weeks of pregnancy during January 1 to March 31 and (ii) participation in postpartum oGTT 6 weeks–12 months after delivery. A total of 76 pregnant women were included in the study (all Caucasian of Czech nationality from South Moravian Region), of those 47 had GDM (those were consenting consecutive subjects positively diagnosed with GDM and then followed from the time of GDM diagnosis till the birth at the Diabetes Centre of the University Hospital Brno) and 29 had physiological pregnancy (those were consenting women who passed midgestational GDM screening with negative result and were followed in several out-patient prenatal centres in the city of Brno until delivery). All participants completed questionnaires mapping vitamin and mineral supplementation during pregnancy. Study participants were not reporting vitamin D or multivitamin supplementation on top of routinely recommended folic acid supplementation. Exclusion criteria were diabetes mellitus type 1 or 2 before pregnancy, non-Caucasian, foreign nationality, multiple pregnancies, and severe comorbidities. Therapy for GDM consisted of diet (100%) and insulin therapy (27.7%).

GDM screening was carried out using oGTT with 75 g of glucose performed between 24th and 30th weeks of pregnancy. GDM diagnosis was established according to the WHO criteria recommended by the Czech Diabetes Society at that time of recruitment (2012): FPG ≥ 5.6 mmol/L, 1 hr after load glucose ≥ 8.9 mmol/L, and 2 hr after load glucose ≥ 7.7 mmol/L (any one of the three above cut-off values qualified for the GDM diagnosis). Postpartum diagnosis of diabetes/prediabetes was based on the WHO criteria for nonpregnant subjects: FPG ≥ 7 mmol/L alone or 2 hr after load glucose ≥ 11.1 mmol/L for diabetes mellitus and FPG 5.6–6.9 mmol/L or 2 hr after load glucose 7.8–11.0 mmol/L for prediabetes.

Study was approved by the Ethical Committee of Faculty of Medicine, Masaryk University, and was conducted in accordance with Helsinki declaration. Each participant provided informed consent. The paper complies with EQUATOR (Enhancing the QUAlity and Transparency Of health Research) network's guidelines.

### 2.2. Blood Samples and 25-Hydroxyvitamin D Measurement

Samples of peripheral EDTA-blood were taken from each participant between 24th and 30th weeks of pregnancy during their scheduled visit in prenatal centre by gynaecologist in non-GDM subjects or by diabetologist during their first visit of diabetes centre and repeatedly 6 weeks–12 months postpartum ibid. Plasma was separated by centrifugation (2 000 g, 10 min, 4°C) and stored at −70°C until analysis. 25(OH)D was measured using an* in vitro* diagnostic enzyme immunoassay kit 25-Hydroxy Vitamin D^S^ EIA (Immunodiagnostic Systems, Boldon, United Kingdom) according to the manufacturer's instructions and using a microtiter plate reader Spectramax 340PC (Molecular Devices, Sunnyvale, California, USA).

### 2.3. Statistics

Data are expressed as medians and interquartile ranges (IQR) or proportions for between-group comparisons. Nonparametric tests were used for comparison between and within the groups (Mann-Whitney and Wilcoxon tests, resp.). Fischer's exact test was used for contingency tables. Correlations were computed using Spearman's correlation coefficients. Software Statistica (StatSoft, Tulsa, Oklahoma, USA) was used for all analyses. *P* < 0.05 was considered statistically significant. Due to the specific requirements of the study—seasonally limited sampling of consecutive GDM and controls subjects—power analysis was performed* post hoc*. The power of the study to detect difference in 25(OH)D levels with given sample size was 0.91 (two means *t*-test).

## 3. Results

Characteristics of study subjects in both groups are shown in Table 1. Positive history of previous GDM was significantly more frequent in GDM group compared to controls (*P* = 0.0491, Fischer's exact test) with no previous foetal macrosomia reported and the same was true for positive family history of any form of DM (*P* = 0.0018, Fisher's exact test). Women with GDM were not significantly older but they were significantly heavier, they had smaller weight gain during pregnancy, and their offspring had significantly lower birth weight. Therefore, we first assessed correlations between 25(OH)D levels in pregnancy and pregestational BMI (*r* = −0.35, *P* = 0.0019), midgestational BMI (*r* = −0.30, *P* = 0.0075), FPG (*r* = −0.36, *P* = 0.0014), postload oGTT values (*P* = NS), weight gain during pregnancy (*r* = 0.35, *P* = 0.0017), and offspring birth weight (*P* = NS). Furthermore, we assessed correlations between postpartum 25(OH)D levels and weight gain during pregnancy (*P* = NS), offspring birth weight (*P* = NS), and parameters of glucose tolerance after delivery, where significant negative correlation with 2 hr after load glucose postpartum was ascertained (*r* = −0.43, *P* = 0.0051). All reported correlations are summarised in Table 2.

**Figure 1:**
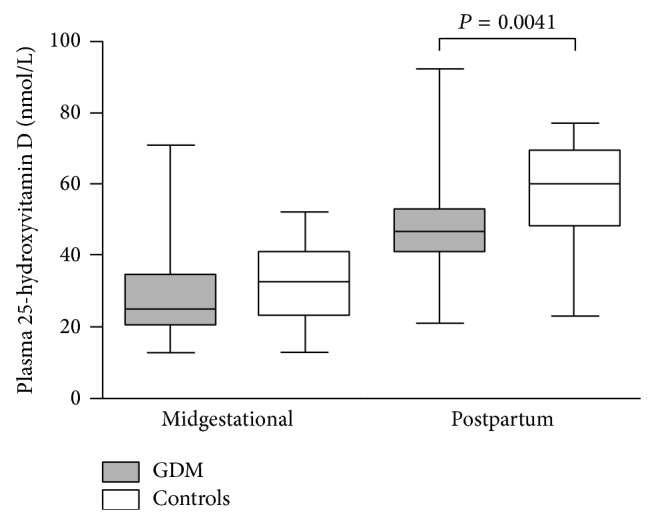
Plasma 25(OH)D levels. Box and Whisker plots were constructed as medians, minimum, and maximum values and IQR. Differences in 25(OH)D levels between midgestational and postpartum values in each group were significant (both *P* < 5 × 10^−6^, Wilcoxon paired test, not shown in the graph).

In spite of the previously assessed inverse relationship of 25(OH)D with BMI, midgestational 25(OH)D levels—both unadjusted and adjusted for midgestational BMI—did not significantly differ between pregnant women with GDM (generally heavier) and healthy controls (*P* = NS, Mann-Whitney). While postpartum 25(OH)D levels raised significantly in both groups (*P* < 1 × 10^−6^ and *P* = 3 × 10^−6^, resp., Wilcoxon test), postpartum 25(OH)D levels in women with GDM history remained significantly lower compared to controls (*P* = 0.0041, Mann-Whitney); see Figure 1.

Based on the results of oGTT repeated up to maximum 12 months postpartum the glucose abnormality was detected in 7 women (14.9%) with history of GDM. We compared both midgestational and postpartum 25(OH)D levels between GDM women with persistent postpartum glucose abnormality and those whose glucose tolerance returned to normal after delivery to test eventual predictive or pathogenic potential of 25(OH)D measurement. There were no statistically significant differences (both *P* > 0.05, Mann-Whitney); however, comparison suffers from rather disparate numbers in the groups (7 versus 40).

Even though there is no consensus on physiological 25(OH)D range, most papers consider levels < 50 nmol/L as deficient [8]. Levels in the range 50–72.5 nmol/L indicate relative insufficiency and levels > 72.5 mmol/L are considered sufficient [7, 32]. In our study, we have found that midgestational vitamin D deficiency (i.e., 25(OH)D levels < 50 nmol/L) was present in majority of the study sample, that is, 45 of 47 (95.7%) women with GDM and 27 of 29 (93.1%) healthy pregnant women (*P* = NS, Fisher's exact test). After delivery, 30 of 47 (63.8%) women with GDM and 10 of 29 (34.5%) controls remained deficient (*P* = 0.012, Fischer's exact test), although majority of blood samples postpartum were taken in summer.

Finally, due to the fact that in April of 2014 Czech Diabetes Society adopted new diagnostic criteria for GDM in accordance with the International Association of the Diabetes and Pregnancy Study Groups (IADPSG) recommendations [33] we reclassified our study sample according to the IADPSG criteria with the following thresholds: FPG ≥ 5.1 mmol/L, 1 hr after load glucose: ≥10.0 mmol/L, and 2 hr after load glucose: ≥8.5 mmol/L retrospectively. Using newly adopted criteria, 24 women would be diagnosed as having GDM and 52 as healthy subjects (note all previously classified controls remained, 23 of previously diagnosed GDM subjects became controls), and then we compared midgestational and postpartum 25(OH)D levels. Interestingly, we found statistically significant differences in both unadjusted and BMI-adjusted midgestational 25(OH)D levels between women with GDM and controls classified by IADPSG criteria (*P* = 0.014 and *P* = 0.006, resp., Mann-Whitney) and also in postpartum 25(OH)D levels between the two groups (*P* = 0.018, Mann-Whitney). In all comparisons 25(OH)D levels in GDM group were significantly lower (data not shown).

## 4. Discussion

Vitamin D seems to have several extraskeletal functions including regulation of glucose metabolism through influencing insulin sensitivity, although the mechanisms are not fully understood. The pancreatic *β* cells express both vitamin D receptor and enzyme 1*α*-hydroxylase which enables them to produce 1,25(OH)_2_D locally [17]. The effect of vitamin D on regulation of pancreatic *β* cell function and insulin secretion could be mediated through intracellular changes in calcium pool. Vitamin D could also enhance insulin sensitivity by stimulating insulin receptor gene expression thereby enhancing insulin mediated glucose transport [34]. In addition, vitamin D may also be needed to ensure a normal rate of calcium flux across cell membranes and maintenance of an adequate cytosolic calcium pool, which is important for insulin-mediated intracellular signalling in insulin-responsive tissues [35]. Finally, several studies suggest that vitamin D could play a role in the pathogenesis of diabetes mellitus type 2 by affecting insulin sensitivity of *β* cell function [36, 37]. Vitamin D is also essential for proper foetal programming and its deficiency during pregnancy may lead to low birth weight and increased susceptibility to chronic disease later in life [38].

Although there is no general consensus on the criteria for vitamin D deficiency in pregnant women, in our study we have found high prevalence of deficiency (when using cut-off < 50 nmol/L) in overall study sample: 95.7% of women with GDM and 93.1% of controls were vitamin D deficient during pregnancy. Reassessment up to 12 months postpartum revealed persisting 25(OH)D deficiency in 63.8% of women with GDM history and 34.5% of controls. Dovnik et al. [39] described seasonal variation of 25(OH)D levels in women of the same stage of pregnancy in Slovenia. Nearly 50% of pregnant women were vitamin D deficient in September while it was 82% in December. This fact could explain the significant difference between postpartum (blood drawn in summer) and low pregnancy 25(OH)D levels in our population which was found in both women with GDM and in controls.

Studies that measured 25(OH)D levels in different time points during pregnancy and after delivery in healthy women provided contradictory results. Holmes et al. [23] have shown that vitamin D deficiency (≤50 nmol/L) in Caucasian population (Irish women) can occur in 95% of pregnant women in 12th week of pregnancy, in 90% in 20th week, in 66% in 35th week, and in 15% 3 days postpartum. Concentrations of 25(OH)D increased in each measurement, being highest after delivery, which could be explained by season in which samples were collected (i.e., mostly during autumn). On the contrary, Haliloglu et al. [40] measured 25(OH)D levels in healthy pregnant Turkish women in each trimester and 6 weeks after delivery and reported that 25(OH)D concentration decreased significantly in each trimester being the lowest postpartum. Contradictory results could be explained by seasonal, geographical, or ethnic vitamin D variation; the paper does not unfortunately mention in what season women were included in the study.

Given the high overall prevalence of vitamin D deficiency, we did not find any statistically significant difference in 25(OH)D levels between woman with GDM diagnosed by WHO criteria and controls during pregnancy. Maghbooli et al. [25] reported higher prevalence of severe vitamin D deficiency (≤12.5 nmol/L) in GDM than in normoglycaemic pregnancies in 741 Iranian women. Nevertheless, vitamin D levels in Asian population are in general lower than in Caucasian population and criteria used in her study would classify most European women as having optimal vitamin D status. Zuhur et al. [41] described significantly lower 25(OH)D levels in 234 Turkish pregnant women with GDM compared to 162 controls. An increased risk of GDM was present only in subgroup with severe 25(OH)D deficiency (<12.5 nmol/L) after controlling for maternal age, previous history of GDM, familiar history of diabetes mellitus type 2, and pregestational BMI. Study of Burris et al. [26] found an inverse association between second trimester 25(OH)D levels < 25 nmol/L and 1 hr after load glucose (50 g) levels; however, only 5% of studied women developed GDM and as mentioned above threshold for vitamin D deficiency differed from our study. Soheilykhah et al. [28] reported that prevalence of vitamin D deficiency is higher among women with impaired glucose tolerance or GDM in 204 Iranian women but they did not find correlation with BMI or FPG. Clifton-Bligh et al. [30] found significantly lower 25(OH)D levels in women with GDM than in healthy pregnant women in the group of 307 Australian women; however, when 4 ethnic subgroups were analysed separately, no association was confirmed between 25(OH)D levels and GDM. Our results are in agreement with other published studies reporting lack of association between vitamin D levels in pregnancy and GDM. For example Makgoba et al. [42] found no statistically significant difference in maternal 25(OH)D levels between GDM and control group in first trimester in 248 British women and Farrant et al. [43] did not find association between maternal vitamin D status in 559 nondiabetic pregnant women from South India and the risk of GDM.

Furthermore, the present study replicated findings of inverse correlation between 25(OH)D and FPG during pregnancy and lack of correlation with age ascertained by others [25, 30, 31]. We also found inverse correlation between 25(OH)D and 2 hr after load glucose after delivery. Contrary to published data [28, 43], we found significant negative correlation between 25(OH)D levels and pregestational and midgestational BMI. Zhang et al. [35] found negative correlation between 25(OH)D levels and pregestational BMI earlier in pregnancy (16th week). Interestingly, we have found positive correlation between total weight increment during pregnancy and midgestational 25(OH)D levels. The findings that GDM women are generally heavier but have lower weight increment during pregnancy and lower offspring birth weight could be explained by the effect of a stricter dietary regime in GDM subjects.

Interestingly, the results appear criteria-dependent and this might be a critical issue in all available studies so far. When applying IADPSG diagnostic criteria for GDM to the same study sample there are statistically significant differences in 25(OH)D levels between women with GDM and those with normoglycaemia not only postpartum but also in midtrimester of pregnancy. Still, since the IADPSG criteria were applied* post hoc*, these results have to be considered hypothesis driving and conclusions speculative.

Several studies investigated vitamin D supplementation in women with GDM [23, 31]. Lau et al. [31] studied whether vitamin D supplementation may improve glycaemic control in women with GDM. Despite the fact that 147 Australian women with GDM were advised to take daily prenatal multivitamins containing 400 IU or 500 IU vitamin D, 41% of the participants had vitamin D deficiency. Asemi et al. [44] assessed the effect of calcium and vitamin D cosupplementation on GDM in a randomised placebo-controlled study (56 Iranian women with GDM) and they observed a significant reduction in FPG, serum insulin levels, and HOMA-IR and increase in QUICKI compared with placebo, and also an increase in glutathione and a reduction in serum LDL-cholesterol and total cholesterol and a significant elevation in HDL-cholesterol were common. Rudnicki and Mølsted-Pedersen [45] reported that supplementation with an active form of vitamin D (1,25(OH)_2_D) was associated with significant decrease of plasma glucose level and possible effect on insulin sensitivity.

Finally, there are certainly limitations of the current study: first of all, a relatively small sample size. For the sake of homogeneity, the enrolment into the study spanned only one quarter of the whole year with the aim to eliminate a possible seasonal effect on vitamin D levels. Moreover, due to the low compliance of GDM women in postpartum screening the sample size was reduced further. According to published data reviewed in [46, 47] only about 50% of women with GDM return after delivery to repeat recommended oGTT and this applied to our study stays in approximately same proportion. As for healthy pregnant women, their participation in postpartum oGTT was entirely voluntary and this resulted in even smaller number of control subjects.

## 5. Conclusions

Our study in pregnant women of central European population did not replicate sporadic previous findings of significantly decreased levels of vitamin D in GDM pregnancy; however, results seem to be criteria-sensitive (WHO versus IADPSG) and the topic warrants further study. We confirmed overall high prevalence of vitamin D deficiency in pregnant women in spite of the GDM presence. The novel and most striking observations of the current study are significantly lower absolute 25(OH)D levels together with significantly higher prevalence of early postpartum 25(OH)D deficiency in women with GDM history compared to those without. Potentially beneficial effect of vitamin D supplementation and the plausible pathogenic role of 25(OH)D deficiency in the subsequent development of diabetes mellitus type 2 in women with GDM history has to be further explored considering the role of vitamin D in modulating insulin sensitivity and glucose metabolism.

## Figures and Tables

**Table 1:** Characteristics of study subjects.

Parameters	GDM (*n* = 47)	Controls (*n* = 29)	*P*
Pregestational parameters			
Age (years)	33 [28–35]	31 [28–33]	NS
BMI (kg/m^2^)	24.45 [22.68–28.91]	21.11 [20.44–24.77]	0.014
History of previous GDM	12.8%	0%	0.0491
Family history of DM	78.7%	17.2%	0.0018
Midgestational parameters (24–30th weeks of gestation)			
FPG (mmol/L)	4.8 [4.5–5.2]	4.1 [4.0–4.4]	<0.001
1 hr after load glucose (mmol/L)	9.2 [8.3–9.6]	5.9 [5.3–6.5]	<0.001
2 hr after load glucose (mmol/L)	8.0 [7.7–8.9]	5.3 [4.8–5.8]	<0.001
BMI (kg/m^2^)	27.16 [25.24–31.25]	23.53 [22.72–29.00]	0.019
25(OH)D (nmol/L)	28.5 [21.0–34.0]	31.7 [24.0–40.0]	NS
25(OH)D < 50 nmol/L	95.7%	93.1%	NS
Postpartum parameters (6 weeks–12 months after delivery)			
Weight gain during pregnancy (kg)	8.0 [5.0–10.5]	14.0 [11.0–17.0]	<0.001
Offspring birth weight (g)	3060 [2750–3480]	3400 [3100–3740]	0.016
Persisting glucose abnormality	14.9%	—	—
25(OH)D (nmol/L)	47.5 [40.0–53.0]	56.5 [48.0–69.0]	0.0041
25(OH)D < 50 nmol/L	63.8%	34.5%	0.012

Data expressed as a median [IQR] or proportions. Differences evaluated by nonparametric Mann-Whitney or Fischer's exact test, respectively.

**Table 2:** Correlations between 25(OH)D levels and selected anthropometric and biochemical parameters.

Parameters	Midgestational 25(OH)D levels		Postpartum 25(OH)D levels	
	*r*	*P*	*r*	*P*
Pregestational BMI	−0.35	0.002	−0.25	0.030
Midgestational BMI	−0.30	0.008	−0.23	0.049
FPG	−0.36	0.001	−0.18	NS
1 hr after load glucose	−0.14	NS	−0.05	NS
2 hr after load glucose	−0.15	NS	−0.43	0.005
Weight gain during pregnancy	0.35	0.002	0.15	NS
Offspring birth weight	0.01	NS	−0.04	NS

Correlations were assessed using Spearman's correlation coefficient. The midgestational 25(OH)D levels were correlated with results of oGTT provided in the midtrimester, and the postpartum 25(OH)D levels were correlated with results of oGTT provided 6 weeks to 12 months postpartum.
